# Potential impact of ocean circulation on the declining Japanese eel catches

**DOI:** 10.1038/s41598-018-23820-6

**Published:** 2018-04-03

**Authors:** Yu-Lin K. Chang, Yasumasa Miyazawa, Michael J. Miller, Katsumi Tsukamoto

**Affiliations:** 1 Application Laboratory, Japan Agency for Marine-Earth Science and Technology, Yokohama, 236-0001 Japan; 2 Department of Marine Science and Resources, College of Bioresource Sciences, Nihon University, Fujisawa, Kanagawa 252-0880 Japan

## Abstract

Recruitment of Japanese eels, *Anguilla japonica*, has declined in recent decades possibly due to both anthropogenic and ocean-atmosphere factors. The potential impact of ocean circulation on the decreasing Japanese eel catches in the western North Pacific was examined based on a three-dimensional particle-tracking method, in which virtual larvae (v-larvae) were programmed to swim horizontally and vertically, in addition to being transported by ocean currents after being released in their North Equatorial Current (NEC) spawning area. Transport patterns varied among years between 1993 and 2013, and dispersion of v-larvae towards East Asia decreased in the last two decades, especially for the western Taiwan and Japan regions. In recent years, instead of entering the Kuroshio and moving towards East Asia as in the 1990s’, more v-larvae tended to enter the southern areas due to the weakening of the NEC and strengthening of subsurface southward flow near the spawning area. Changes in ocean circulation in the western Pacific appear to be caused by the weakening of subtropical and tropical wind stress curl in the past two decades. This suggests that decadal changes in ocean circulation have occurred that affect the larval migration success of the Japanese eel to their recruitment areas.

## Introduction

Anguillid eels are widely distributed in the Indo-Pacific and North Atlantic regions where the juveniles live in freshwater and estuarine habitats^1,2^. Their adults migrate offshore to spawn over deep water and their larvae, called leptocephali have a long larval dispersal stage that varies among tropical and temperate species^3–5^. World eel recruitment has significantly declined in the past few decades^6^, raising concerns about what are the major causes. Overfishing and habitat loss due to human activities were considered to be one of the primary reasons causing the eel recruitment decline^7^. Changes in ocean circulation or oceanic conditions may also significantly affect eel recruitment^8–15^, or both types of factors have likely contributed^16^.

The Japanese eel, *Anguilla japonica*, is one of the most important eel species for fisheries and aquaculture, and it has been listed as endangered on the IUCN red list^17^. Among the Northern Hemisphere species of eels that have all experienced declines, the Japanese eel seems to have shown the earliest recruitment declines that started in the 1970s^18^. After about 2010, the annual recruitment had decreased by as much as 90% compared to eel catches in the 1960s^17^.

The Japanese eel is distributed across East Asia in areas that are adjacent to the western Pacific Ocean. Their spawning area is located along the West Mariana Ridge within the westward flowing North Equatorial Current (NEC)^19,20^. The NEC forms a zone of westward flow from about 10–20°N, which then bifurcates into northward flow (the Kuroshio) and southward flow (the Mindanao Current)^21,22^. The eel larvae are carried primarily by the NEC and Kuroshio toward their growth habitats in East Asia, although it is possible that some active swimming occurs^5^. The maturing silver eels migrate thousands of kilometers to return to their spawning area for reproduction^3^. The migration process of Japanese eels, however, is not well understood due to limited direct observational evidence on adult migration^23^ or larval dispersal^24^. Numerical modelling methods, therefore, have been used to simulate the potential migration paths of Japanese eels, and to evaluate their linkages to ocean-atmosphere changes^12,14,15,25,26^.

Previous numerical modelling studies on Japanese eel leptocephali examined the effect of ocean circulation and ocean-atmosphere conditions on larval transport. For example, some studies suggested that the transport of larvae carried by currents from the NEC to the Kuroshio is lowest in the El Niño years compared to the non-El Niño periods^11,12^. Other recent modelling studies evaluated the interannual migration success of Japanese eel larvae from their spawning area to East Asia and suggested that more complex ocean circulation factors related to the NEC, Kuroshio, and eddies can impact the migration, which are connected to the Philippine-Taiwan Oscillations (PTO)^14,15^. The strength and position of the NEC appear to be important factors^14,15,27^, and tropical rainfall affecting the position of the salinity front that can form in the spawning area may also affect recruitment in some years^27^. Therefore, ocean-current and climate-related factors need further investigation using long-term simulations.

The western Pacific Ocean is one of the most variable ocean regions, having the fastest sea level rise in the world^28^. The western North Pacific Ocean is characterized by major oceanic features, such as the NEC, the Kuroshio, the Subtropical Countercurrent (STCC), and eddies, which will likely vary during a changing climate. For example, the Kuroshio has warmed two times faster than the global warming rate in the past century^29^. The NEC bifurcation latitude is shifting southward in the past 60 years^30^. The Kuroshio along the East China Sea has gradually moved onshore^31^, and the Luzon Strait intrusion into the South China Sea is weakening in the last two decades^32^.

The recruitment of the Japanese eel has been declining during these years even after the some of the major anthropogenic impacts have been reduced in at least some areas, so the effects of the changing ocean need further exploration. The present study investigates the potential impact of ocean circulation on the decreasing Japanese eel catches in the western North Pacific. The 22-year (1993–2013) common period for model reanalysis and annual glass eel catches is used. Note that the present work does not strictly obey the definition of the time period required to study climate effects due to the limitation of data. The long-term (1993–2013) dispersal of virtual larvae (v-larvae) in the western Pacific is simulated based on a three-dimensional (3D) particle tracking method, in which swimming behavior is included in addition to transport by ocean currents. Biological factors affecting mortality (i.e., feeding success or predation) are not considered in the present work that focuses on the effects of ocean circulation. In addition to the yearly variation that was reported by the earlier studies, a long-term trend of decreasing dispersal towards parts of East Asia is observed in our simulations. We also tentatively examine the possible causes of recent ocean circulation changes that could be connected to climate changes.

## Data and Methods

### Observation data

Annual Japanese glass eel (recruitment-stage early juveniles) catch data from 1993 to 2013 for Taiwan and Japan were obtained from the Taiwan Fisheries Yearbook (Fisheries Agency, Council of Agriculture, Taiwan) and Japan Aquaculture Information News (The Nihon Yoshoku Shimbun, Tokyo, Japan, which collected the official data from Ministry of Agriculture, Forestry, and Fisheries in Japan), respectively^25^. Ocean drifter data were obtained from the Global Drifter Program (http://www.aoml.noaa.gov/phod/dac/index.php). Surface drifters released in the NEC region from May to September during two periods (1993–1997 and 2009–2013) were selected. There were 17 and 29 drifters in the early and later periods, respectively.

### Reanalysis data

The data-assimilative ocean circulation model known as the Japan Coastal Ocean Predictability Experiment 2 (JCOPE2) provides the three-dimensional currents and hydrological fields that were used in particle tracking in the present study. JCOPE2 was constructed from the Princeton Ocean Model with a generalized coordinate system^33^. The model domain of JCOPE2 encompasses the western North Pacific (10.5–62°N and 108–180°E), with a horizontal resolution of 1/12° (8–9 km) and 46 vertical layers. The external forcing to drive JCOPE2 includes wind stresses and net heat/freshwater fluxes at the sea surface converted from the six-hourly atmospheric reanalysis produced by the National Centers for Environmental Prediction*/*National Center for Atmospheric Research. Satellite and *in situ* temperature and salinity data were assimilated into the model based on a three-dimension variational method^33^. The daily JCOPE2 reanalysis fields cover the period from January 1993 to the present. Comparison of simulated trajectories of passive particles carried by JCOPE2 and observed trajectories was performed in a previous study^14^, which showed the satisfactory performance of JCOPE2 in simulating the 3D circulation over the western North Pacific Ocean.

The wind data used for calculating wind stress curl and Sverdrup transport was based on the National Centers for Environmental Prediction/National Center for Atmospheric Research (NCEP/NCAR) reanalysis products (https://www.esrl.noaa.gov/psd/data/reanalysis/reanalysis.shtml).

### Particle-tracking scheme

A three-dimensional (3D) particle-tracking method was used to simulate the movement of virtual eel larvae (v-larvae). The particles were carried by ocean currents in addition to having their own swimming behavior. The 3D particle-tracking scheme developed by Ohashi and Sheng^34^ was used in this study. The tracking scheme was based on the fourth-order Runge–Kutta method^35^. The tracking time step was three hours. The same tracking scheme was used previously by Chang *et al*.^14,26,36^ for investigating the migration of Japanese eel larvae and adults in the western Pacific Ocean and was also applied to simulations of the long-distance migration of adult American eels in the Atlantic Ocean^37,38^.

Two important swimming behaviors were considered in this study: diel vertical migration (DVM) and horizontal swimming. Eel larvae likely show DVM behavior^39^; i.e., they remain in upper surface waters at night and swim to deeper waters probably to avoid predators during the daytime. The age-dependent DVM and horizontal swimming followed a previous study^14,40^. V-larvae at night stayed at a fixed depth of 50 m. The swimming speed and daytime diving depth linearly increased with age. Swimming speed increased by 0.0006 m s^−1^ per day, and daytime DVM deepened by 0.75 m per day. (i.e., on day 0, swimming speed was zero, and v-larvae did not perform DVM; On day 100, swimming speed was 0.06 m s^−1^, and DVM was between 50 to 125 m). The maximum swimming speed was 0.14 m s^−1^, and the maximum daytime diving depth was 230 m to the end of the simulation. A random walk displacement was included to represent unresolved sub-grid turbulent flow and other local processes^34^. The estimated maximum horizontal and vertical displacements due to the random walk were 600 m and 20 m, respectively. The duration of day and night were determined by the time of sunrise (6 am) and sunset (6 pm) throughout the simulation. The swimming direction of the v-larvae was set to be the same as the local flow in the open ocean with water depths greater than 100 m as in the previous study to allow intercomparison of the results^14^. When the v-larvae approached the coastal and shelf waters with water depths shallower than 100 m, they were set to search for lower salinity and swim toward coastal fresher waters^41^. As actual eel larvae likely do not swim continuously throughout their early life history and may use a directional swimming orientation^5^, an additional simulation using different larval behaviors was conducted as a control to establish that larval behavior was not affecting the overall trend of decreasing transport to East Asia over time (see Discussion section).

### Experimental design

Numerical experiments were conducted to examine the interannual variation of v-larvae transport during a simulation period from 1993 to 2013. The release region and time of v-larvae were chosen on the basis of the observed spawning area and season based on the collections of Japanese eel eggs, newly hatched preleptocephali, and spawning-condition adults along of the southern West Mariana Ridge^3^. The particles (v-larvae) were released at locations spread over the region of 140 to 143°E and 12 to 15°N with a separation distance of 10 km in both zonal and meridional directions, and one being released at each location. Leptocephali estimated to be 10–40 days old were found in June and July^42^. The releases time were set from May 1 to July 31 and each release was staggered by a time interval of 5 days during the three-month period. About 18,000 v-larvae were released each year. The Japanese eels born in summer likely reach the East China Sea and south of Japan in winter and early spring^24^. The migration period estimated from observations is about six to eight months. The tracking duration was set to be eight months to track v-larvae during their migration^14^.

**Figure 1:**
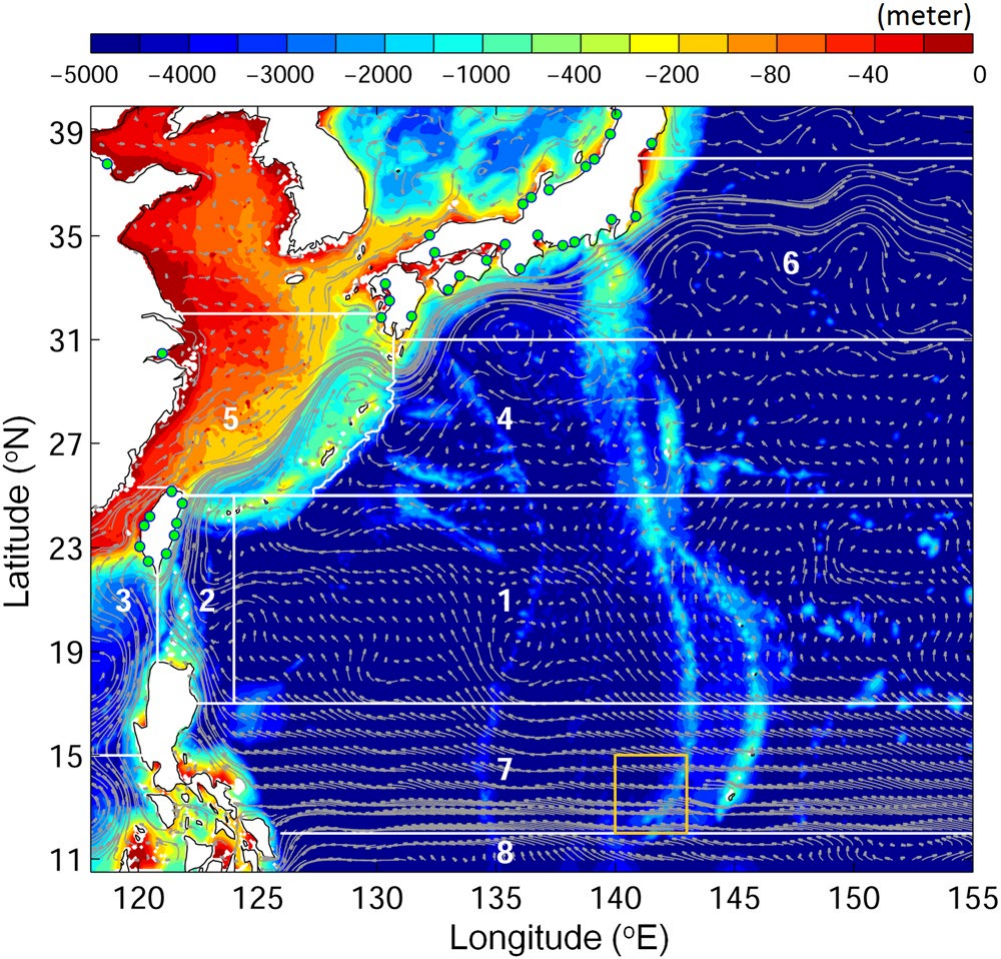
The model sub-regions (labeled 1–8, separated by white lines) shown over the bathymetry (color) in the western North Pacific Ocean. Gray vectors show the average ocean currents in upper 200 m from year 1993 to 2013. The yellow box represents the Japanese eel spawning area. The green dots mark the rivers included in tracking scheme. *Figure is created using MATLAB R2011b* (http://www.mathworks.com/).

The number of v-larvae reaching each of 8 designated regions (Fig. 1) were calculated as visitation frequency. The regions were (1) Subtropical Countercurrent (2) Upstream Kuroshio (3) the South China Sea and Taiwan Strait (4) the Philippine Sea (5) East China Sea and Okinawa Trough (6) South of Japan (7) Northern North Equatorial Current (8) Southern North Equatorial Current. Visitation frequency was defined as the number of v-larvae that visited each individual grid, in which each of the v-larvae would only be counted once on the same grid in each simulation.

The linear trend (percentage per year) for observed glass eel recruitment and simulated v-larvae dispersion referred to the 22-year (1993–2014) mean. The significance of linear trends was examined based on Mann-Kendall tests. Significance was defined as p < 0.05.

### Data Availability

The datasets generated during and/or analysed during the current study are available from the corresponding author on reasonable request.

## Results

**Figure 2:**
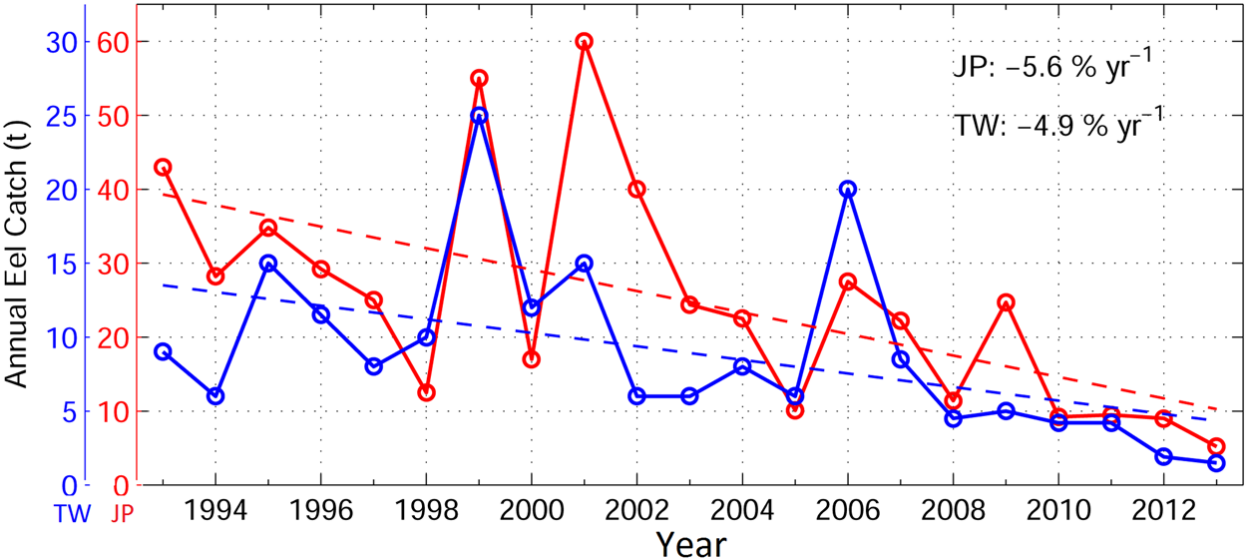
Observed recruitment-stage glass eel catches of the Japanese eel, *Anguilla japonica*, in Japan (red) and Taiwan (blue). Dashed lines show the linear trends (p < 0.01). Unit is in t. *Figure is created using MATLAB R2011b* (http://www.mathworks.com/).

The observed annual glass eel catches in Japan and Taiwan showed inter-annual fluctuations (Fig. 2). The eel catch variations between Taiwan and Japan were significantly correlated (r = 0.69, p < 0.01). Apart from the biological factors, such as changes in demographic dynamics could potentially influence the annual recruitment, the high correlation also suggests the eel catches in Taiwan and Japan are likely influenced by the same environmental factors^25^, such as ocean-atmosphere conditions. In addition to year-to-year variations, a long-term decreasing trend can be seen (p < 0.01). The annual eel catches in Taiwan and Japan showed overall decreases of about 5% per year in the past two decades.

**Figure 3:**
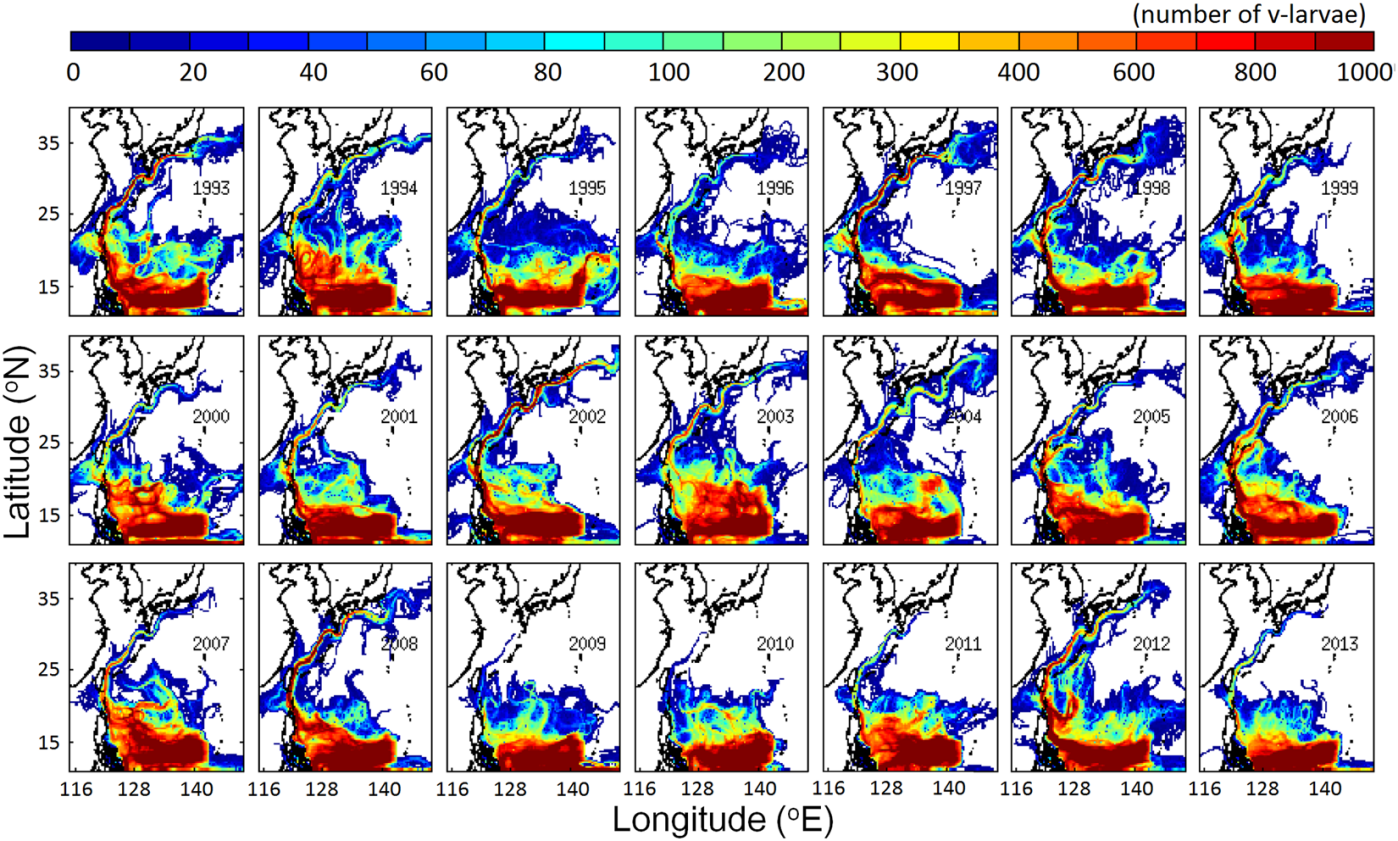
V-larvae visitation frequency from the year 1993 to 2013 after being released in the spawning area. Unit is number of v-larvae. *Figure is created using MATLAB R2011b* (http://www.mathworks.com/).

**Figure 4:**
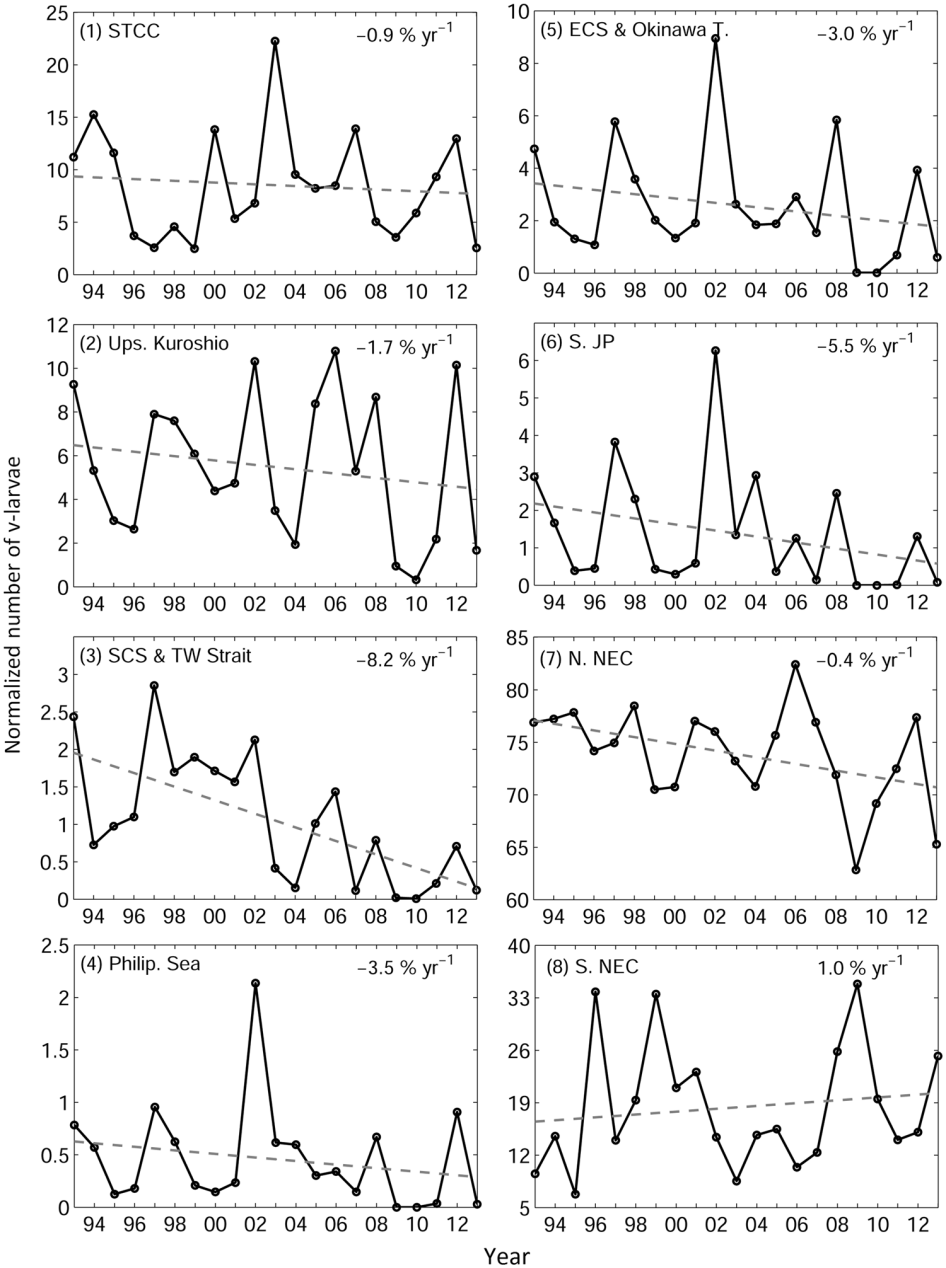
Visitation frequency v-larvae in each sub-region. Dashed lines show the linear trends after being released in the spawning area. 1 normalized unit is 20,000 v-larvae. Percentage numbers indicate the overall rate of change per year. *Figure is created using MATLAB R2011b* (http://www.mathworks.com/).

Simulated v-larvae transport paths were widely distributed in the western North Pacific during each of the 21 simulation years with some clear variations among years (Fig. 3). The greatest visitation frequencies of v-larvae occurred in the NEC where larvae were transported away from their spawning area, but in some years many larvae entered areas north of 15°N. Some v-larvae entered the Kuroshio and arrived to East Asia, but others were advected southward or remained in the NEC region until the end of the 8-month simulations. The amount of v-larvae arriving in the region south of Japan was greatest in 1993, 1997, 2002, 2004, and 2008 and was lowest or none in 2007, 2009, 2010, 2011 and 2013 as well as in some earlier years (Figs 3 and 4). For those v-larvae that reached south of Japan, the main transport routes were sometimes more near-shore (i.e., 2002) or farther away from the coast (i.e., 2004).

Classifications of the v-larvae dispersion by region (Figs 1 and 4) showed that there were decreasing trends of dispersion into many of the sub-regions north of the spawning area (region 1–7, Fig. 4). Due to the alternations between higher and lower numbers of v-larvae entering the regions, the regressions were not significant (p = 0.10–0.78) except for in the South China Sea and Taiwan Strait region (p < 0.01) and the South of Japan region (p = 0.04), both of which have a direct impact on the eel catches in Taiwan and Japan, respectively. In contrast, the number of v-larvae were increasing to the south of the spawning area (region 8) in the past 21 years. As the release number of v-larvae per year was fixed, the changes of v-larvae distributions indicated that v-larvae tended to be advected more southward in recent years in comparison to the earlier decades, resulting in reductions of v-larvae recruitment along the Kuroshio. Cross-correlations of v-larvae visitation frequencies were observed between most sub-regions (Table 1) except the STCC (region 1). V-larvae variation in the STCC was not related to the Kuroshio system but was linked to the NEC. The amount of v-larvae remaining in the STCC region depends on the bifurcation of the NEC as described in a recent study^14^. V-larvae in the Kuroshio associated regions (region 2–6) were all correlated with each other. The v-larvae entry into the areas south of Japan (region 6) was significantly associated with v-larvae variation near Taiwan (region 2 and 3), similar to the relationship observed in glass eel catches (Fig. 2). V-larvae variation in the southern NEC was negatively-correlated with all other sub-regions, suggesting that increases of v-larvae to the south of the spawning area corresponds to decreases of v-larvae to East Asia.

**Table 1:** Cross-correlation between the subregions shown in Fig. 1.

Region	(1)	(2)	(3)	(4)	(5)	(6)	(7)	(8)
(1)	—	*0.03*	*−0.23*	*0.07*	*−0.05*	*−0.05*	*0.32*	*−0.66*
(2)	**0.90**	—	*0.64*	*0.65*	*0.78*	*0.57*	*0.67*	*−0.32*
(3)	**0.32**	**<0.01**	—	*0.51*	*0.64*	*0.56*	*0.45*	*−0.10*
(4)	**0.75**	**<0.01**	**0.02**	—	*0.93*	*0.94*	*0.35*	*−0.32*
(5)	**0.83**	**<0.01**	**<0.01**	**<0.01**	—	*0.91*	*0.41*	*−0.27*
(6)	**0.81**	**<0.01**	**<0.01**	**<0.01**	**<0.01**	—	*0.29*	*−0.30*
(7)	**0.16**	**<0.01**	**0.04**	**0.12**	**0.07**	**0.19**	—	*−0.62*
(8)	**<0.01**	**0.16**	**0.68**	**0.16**	**0.23**	**0.18**	**<0.01**	—

Top right shows the correlation coefficients (italic), and bottom left indicates p-values (bold). Number for regions are (1) Subtropical Countercurrent (2) Upstream Kuroshio (3) the South China Sea and Taiwan Strait (4) the Philippine Sea (5) East China Sea and Okinawa Trough (6) South of Japan (7) Northern North Equatorial Current (8) Southern North Equatorial Current.

**Figure 5:**
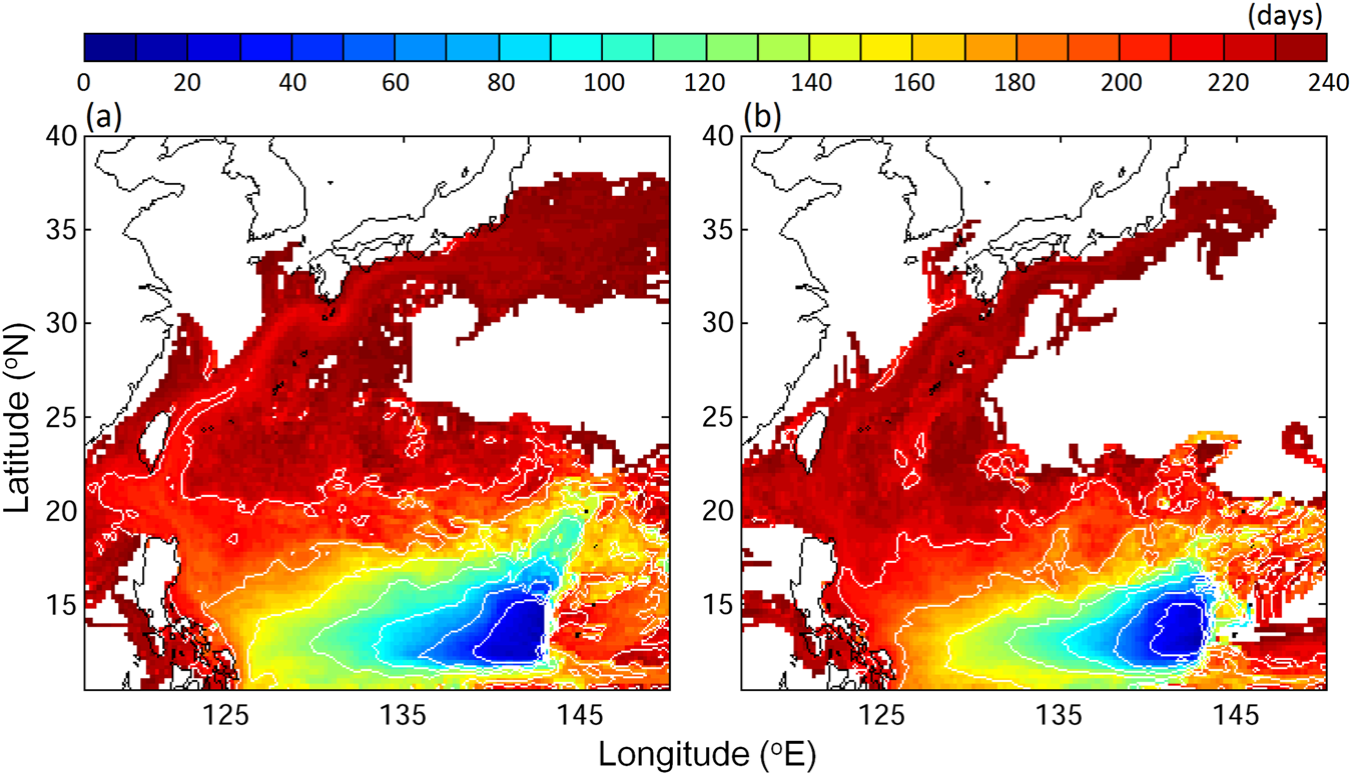
Mean ages of all v-larvae crossing a given grid (days) for the (**a**) years 1993–1997, and (**b**) years 2009–2013 of v-larvae released in the spawning area. *Figure is created using MATLAB R2011b* (http://www.mathworks.com/).

V-larvae dispersion towards East Asia has been reduced in recent years as illustrated by a comparison between 1993–1997 and 2009–2013 (Fig. 5). In the 1990’s, v-larvae were able to reach the East China Sea in 7 months, whereas v-larvae over the past few years could only approach the east coast of the Philippines in that amount of time. The intrusion through Luzon Strait to the South China Sea, as well as the final arrival to areas south of Japan both declined in recent years.

**Figure 6:**
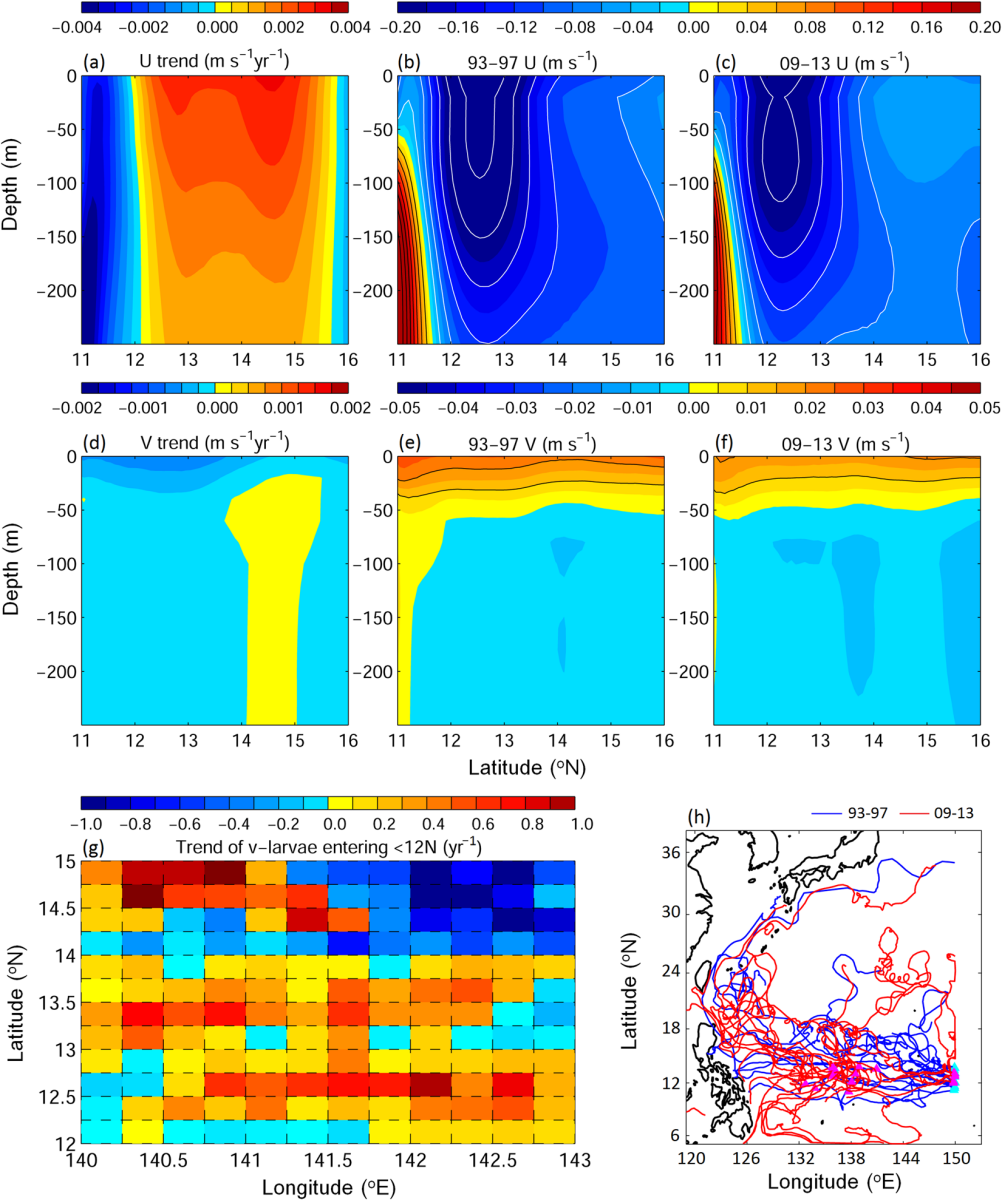
Linear trends from 1993 to 2013 (**a**,**d**) and 5-year averaged vertical profiles (**b**,**c**,**e,f**) in the NEC (130–143°E mean) region of zonal (**a**–**c**) and meridional velocities (**d**–**f**) for the years 1993–1997 (**b**,**e**) and years 2009–2013 (**c**,**f**). Positive and negative values for zonal (meridional) velocity represent eastward (northward) and westward (southward) directions, respectively. The trend for those v-larvae entering southern NEC is shown in (**g**). Positive (negative) trends suggested increase (decrease) of v-larvae entering southern NEC. Observed ocean drifter trajectories in the years 1993–1997 (blue) and years 2009–2013 (red) is shown in (**h**). *Figure is created using MATLAB R2011b* (http://www.mathworks.com/).

The decreases of v-larvae being transported to the margins of East Asia appear to be caused by changes of ocean current patterns. In the past two decades, the westward flow of the northern NEC has been getting weaker (Fig. 6a–c, positive eastward trend between 12–15°N suggested that the westward NEC was weakening), while the subsurface (below 50 m) southward flow south of 14°N was getting stronger (Fig. 6d–f, negative trend south of 14°N indicated more southward flow). The weakening of the NEC would make v-larvae stay in the NEC longer, and the enhanced southward flow, although weak, could effectively bring more v-larvae southward. We traced the origin of the v-larvae entering the southern NEC (Fig. 6g), and many originated from 12–14°N where the enhanced southward current was observed (Fig. 6d). In addition to v-larvae trajectories, the limited numbers of observed drifters showed generally consistent patterns with the changes of v-larvae transport patterns in the past two decades (Fig. 6h). Drifters in the early decade (1993–1997) mostly joined the Kuroshio after departing from the northern NEC, but drifters launched in recent years (2009–2013) partly moved southward without entering the Kuroshio.

**Figure 7:**
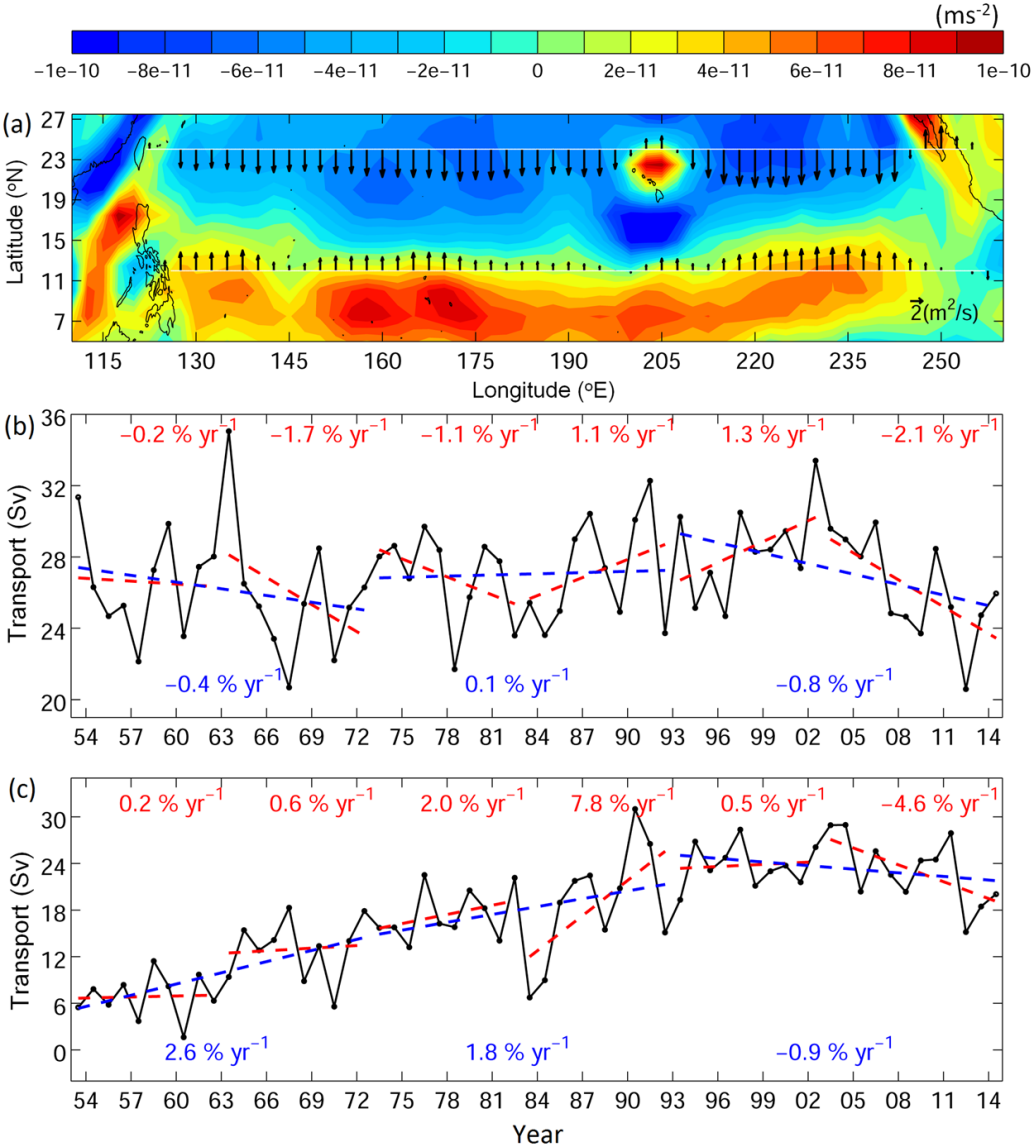
1953–2014 mean (**a**) wind stress curl (color) and Sverdrup transport (black vectors) based on the NCEP reanalysis data. Annual mean integrated Sverdrup transport (Sv, 1 Sv = 10^6^ m^3^s) at (**b**) 24°N and (**c**) 12°N. Red and blue dashed lines are 10-yr and 20-yr linear trends, respectively. Percentages indicate the overall rate of change per year.

The changing ocean circulation in the western North Pacific is likely caused by the forcing of the large-scale winds. The classic theory of Sverdrup dynamics explains the relationship between the wind stress curl (WSC) and the western boundary current (i.e., strengthening of WSC leads to the enhancement of western boundary current)^43^. The dipole of WSCs exist in the subtropical and tropical Pacific, with anti-cyclonic WSC to the north of 15°N and cyclonic WSC to the south of 15°N (Fig. 7a). The corresponding southward Sverdrup transports in the subtropical Pacific, and northward Sverdrup transports in the tropical Pacific then merge and form the western boundary currents that are the Kuroshio and the Mindanao Current. Decadal variations appeared in both subtropical and tropical WSC and the corresponding Sverdrup transports in the past 60 years (Fig. 7b,c). The subtropical and tropical WSC weakened in the previous two decades, the corresponding weakening of Sverdrup transport lead to the reduction of ocean interior currents, which contributed to the weakening of the NEC, and the weakening of subtropical WSC also led to the weakening of the Kuroshio. The weakening of northward Sverdrup transport in the tropical Pacific could serve as a candidate, explaining the stronger southward flow in the subsurface water south of 14°N if the vertical shear was fixed (i.e., the weakening of vertically integrated northward transport would result in weaker northward current near the surface and stronger southward current in the subsurface, Fig. 6e,f). The strengthening of subsurface southward flow could also occur with the change of vertical shear, in order to balance with the change of the horizontal density field^43^. Overall, the weakening of the NEC together with the strengthening of southward subsurface current could have resulted in greater transport of v-larvae to the areas south of the spawning area in recent years. Furthermore, the weakening of the Kuroshio in the recent decade also contributed to reduction of v-larvae northward dispersion. The Kuroshio along the East China Sea also appears to be weakening in association with the changes of wind stress curl at 20–30°N^44^, which also explains why the decreasing rate of v-larvae in the downstream Kuroshio region (Fig. 4, South of Japan region 6) is greater than that in the upstream Kuroshio (Fig. 4, region 2). As a result, v-larvae dispersions in recent years were confined further to the south in comparison to the earlier decade. Apart from the Kuroshio, glass eel catches in Taiwan are also affected by the intrusion of water through the Luzon Strait and into the Taiwan Strait. While the subtropical and tropical WSCs weakened, the Luzon Strait intrusion also decreased due to the weakening of westward current and reduced eddy activity^45^. The weakening of Luzon Strait intrusion together with the weakening of the Kuroshio likely leads to decreases in eel catches in Taiwan.

## Discussion

The present study explored the potential impact of western North Pacific Ocean circulation on the declining and fluctuating Japanese eel catches in East Asia based on a three-dimensional particle-tracking method, in which v-larvae were programmed to swim (horizontal swimming and vertical DVM), in addition to movements resulting from transport by ocean currents. The simulated v-larvae dispersion to different regions of the western North Pacific showed clear variations among years, and in recent decades there was a decrease in the number of v-larvae reaching areas near Japan and Taiwan where the species recruits. Previous larval transport modelling studies have found similar interannual variability and that ocean-atmosphere factors such as El Niño or the Philippines–Taiwan Oscillation (PTO) appear to influence the number of larvae that may successfully reach their recruitment areas^12,14,15^. Those studies and the present study examined the importance of ocean circulation to Japanese eel larvae dispersion, however we focused on the possible effect of changing winds. Our findings further suggest that changes in ocean circulation could be playing a role in the decreasing trend of glass eel catches in Japan and Taiwan using long-term simulation results. An important factor in the decreases in recruitment could be that instead of entering the Kuroshio and moving northward, more larvae may be moved southward due to weakening of the westward flow of the NEC or a southward shifting of its position and strengthening of subsurface southward flow near the spawning area in recent years. These changes in ocean circulation may be in response to changes in winds resulting from a weakening of subtropical and tropical wind stress curl in the past two decades. Other factors, such as changes in horizontal temperature or salinity balanced by the changes of vertical shear may also lead to the strengthening of subsurface southward current. A detailed dynamical analysis is necessary for understanding the actual cause for change of ocean circulation.

The weakening of wind stress curl was proposed to be connected to global warming^46^, and climate projections have also suggested that weakening of the NEC and the upstream Kuroshio (south of 25°N) could be associated with the increase of greenhouse gases^47^. However, the actual dynamical forcing and responses remain unclear, and uncertainty also exists within different models^47^. Moreover, the global temperature warmed up rapidly starting in the 1980′s, yet the wind stress curl showed decadal oscillations prior to the 1990’s. The 22-years of ocean reanalysis available for our study could be too short to fully represent all aspects of the long-term climate variation, therefore, the role of the warming climate in changing atmospheric conditions that influence ocean circulation requires more exploration.

Although the present study and previous transport modelling studies illustrate the possible role of ocean circulation in causing distinct variability in the transport patterns that could affect recruitment levels, there are other factors that could be involved in the actual levels of recruitment each year. For example, the general decreasing trends of the time-series of glass eel catches in Taiwan and Japan were similar to the number of larvae reaching those areas in the simulations, but their year by year variations were not significantly correlated (p > 0.1). Other factors that could be involved include the number of adult eels migrating to the spawning area each year, which has the potential to be influenced by climatic factors affecting the freshwater habitats where the juveniles live^48^. That could affect the number of larvae being transported out of the spawning area. The fishing effort could also vary year by year, which could potentially influence the amount of annual recruitment. Another factor that appears to be unique for the Japanese eel is that the latitude of their spawning locations appear to be influenced by the latitude of a shallow salinity front that often forms in the NEC as a result of tropical rainfall^42,49,50^. When a distinct front is present, spawning seems to occur south of the front, but when there is no front, the spawning occurs over a wider range of latitude^51^. It is also possible that changes in productivity might affect early larval survival through reductions in their marine snow food source^16^. Changes in productivity-related indicators have changed in the NEC region^52,53^, and chlorophyll-a has steadily decreased and surface temperatures have increased in the North Pacific subtropical gyre since 1998^54^. Distinguishing the possible contributions of these types of biologically linked factors and the effects of changing currents will require further detailed research efforts.

**Figure 8:**
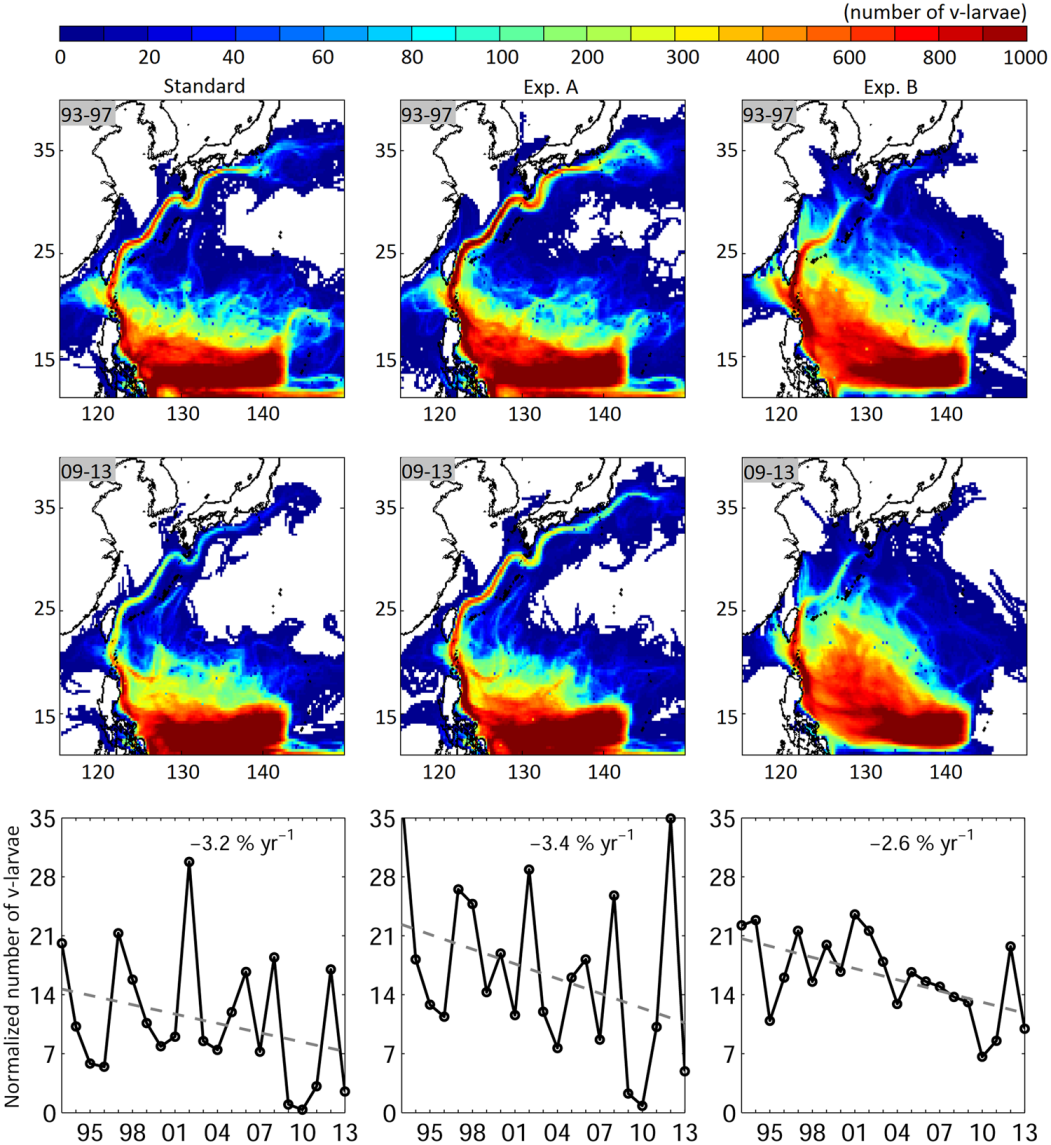
V-larvae visitation frequency for the years (top) 1993–1997 and (bottom) 2009–2013, and the visitation frequency in the regions adjacent to East Asia (sub-regions 2–6) for the (left) standard experiment, (middle) Exp. A and (right) Exp. B. 1 normalized unit is 20,000 v-larvae. Figure is created using MATLAB R2011b (http://www.mathworks.com/).

Another biological factor that could be more important than previously considered is that of active swimming by the larvae, whose bodies are filled with energy storage material that could be used for swimming^5^. In order to confirm that the general findings of dispersion decreases were not sensitive to the particular swimming strategy used in the simulations, different swimming behaviors were examined. Considering that the 24-hour swimming used in the present study and previous ones^14,26^ could be energy consuming, and therefore not biologically accurate, and the newborn larvae may just drift with ocean currents while they are feeding and growing, an additional experiment (Exp. A) set v-larvae to start swimming when they reached 30-days old, and they only swam at night when they probably stop feeding. The linearly increasing swimming speed, DVM and swimming direction were the same as the standard simulation run. Another experiment (Exp. B) used the same strategy as Exp. A, but set a fixed swimming direction of northwestward for v-larvae to facilitate movement towards and across the Kuroshio. Both experiments showed decreasing trends of reaching areas near East Asia (Fig. 8), especially with northwest swimming (p < 0.01), which also showed less interannual variability, less southward transport, and higher numbers of v-larvae crossing out of the Kuroshio system and approaching their recruitment areas. This suggests the decreasing dispersion caused by the ocean circulation changes is robust and that larval behavior is important. The result also suggests that new research on the influence of directional swimming in biologically realistic contexts will be useful in future studies.

Further research on the effects of ocean current changes and the influence of larval behaviors are needed not only for the Japanese eel, but also on other species, such as *A. marmorata, A. bicolor pacifica, and A. luzonensis* in the western Pacific as well as the eel species in the other subtropical gyres where equatorial currents and western boundary currents are important for transporting larvae^5^. How changes in the ocean circulation or in biological factors are influencing larval survival will be essential to help efforts to conserve these interesting species in a changing ocean-atmosphere system in the future.
